# A preliminary investigation into self-compassion and compassion-based intervention for mental health in the performing arts

**DOI:** 10.3389/fpsyg.2025.1512114

**Published:** 2025-02-06

**Authors:** Courtney C. Walton, James N. Kirby, Sabrina McKenzie, Caroline X. Gao, Rosemary Purcell, Simon M. Rice, Margaret S. Osborne

**Affiliations:** ^1^Melbourne School of Psychological Sciences, The University of Melbourne, Melbourne, VIC, Australia; ^2^School of Psychology, University of Queensland, Brisbane, QLD, Australia; ^3^Melbourne Conservatorium of Music, The University of Melbourne, Melbourne, VIC, Australia; ^4^Orygen, Melbourne, VIC, Australia; ^5^Centre for Youth Mental Health, The University of Melbourne, Melbourne, VIC, Australia

**Keywords:** musicians, actors, dancers, wellbeing, meditation, mixed-methods

## Abstract

**Objectives:**

Very little is known about the role of self-compassion on performing artists’ mental health. This project had two primary aims. First, was to examine the relationship between self-compassion and mental health among performing artists in dance, music, and acting. Second, was to test a brief compassion-based intervention to establish proof of concept for future work.

**Method:**

Two sequential studies recruiting Australian performing artists were conducted. In Study 1, a cross-sectional online survey of performing artists explored patterns of association between self-compassion and mental health. In Study 2, participants engaged with a brief compassion-based intervention including an online workshop and daily meditation over 3 weeks. Descriptive pre-post data combined with thematic analysis of semi-structured interview transcripts identified performer perspectives of the compassion-based intervention.

**Results:**

In Study 1, 211 participants were included in the final cross-sectional analysis, which showed that the intention to be self-compassionate was associated with lower symptoms of depression and anxiety, and higher wellbeing, even while controlling for several key demographic and risk factors (stress, alcohol use, and body appreciation). In Study 2, ten participants completed the intervention with medium-large improvements in self-compassion and small improvements in wellbeing and body appreciation. Qualitative data demonstrated that participants experienced self-compassion to be helpful, however fears of lowering standards existed. Participants feedback from this study can now directly inform future compassion-based interventions among performers.

**Conclusion:**

While acknowledging the small sample size, these findings suggest that self-compassion may help performing artists manage various forms of internal, interpersonal, and environmental distress. These findings warrant progression to controlled evaluation of compassion-based interventions within larger samples of performing artists.

## 1 Introduction

Performing artists such as actors, musicians, and dancers are exposed to many stressors which can impair wellbeing. These include pressures around competition and comparison, job instability, financial insecurity, body appearance, and injury (Willis et al., 2019). However, limited research has focused on specific psychological approaches to supporting individuals in such challenging contexts, which hampers understanding of risk within this population and potential approaches to managing mental ill-health. The goal of this project was to obtain a better understanding as to how compassion-based interventions (CBIs) could play a critical role in supporting performing artists.

The few studies that have explored the mental health of performing artists – especially musicians and dancers – have reported concerning rates of mental ill-health including symptomatology associated with depression, anxiety, eating disorders, and psychological distress (Dwarika and Haraldsen, 2023; Kegelaers et al., 2021; Kenny et al., 2014; Loveday et al., 2022; Willis et al., 2019). For example, in a large Norwegian sample of musicians, Vaag et al. (2016) found evidence for increased psychological distress, and more than double the prevalence of both anxiety and depression as compared to population norms. In a study of professional dancers, one in five reported clinically significant symptoms of anxiety, depression or eating disorders, with a quarter describing a subjective need for psychotherapy (Junge and Hauschild, 2023). The increased rates of mental health seen across both professional and student performing artists is thought to reflect exposure to a range of physical, psychological, interpersonal and environmental risks (Noh et al., 2009).

Body image disturbance has long been established as a key risk factor for a range of negative physical and psychological outcomes among performing artists, especially dancers. For example, in a qualitative study of occupational stress among dancers in Korea, a range of physical, psychological, interpersonal, and situational factors were found to be key sources of stress (Noh et al., 2009). The most common source of stress related to physical appearance and condition, such as maintaining a certain body shape or weight. Among those performing in dance, more frequent attention has been paid to body image concerns and eating disorders, with one meta-analysis showing dancers had a three-fold higher risk of eating disorder than the general population (Arcelus et al., 2014).

Performing artists also experience high levels of stress, which can stem from various sources. Several studies have documented the role of musculoskeletal injuries among both musicians (Elam et al., 2022) and dancers (Vassallo et al., 2019), in addition to performing artists’ low satisfaction with both financial reimbursement (Kenny et al., 2016) and job security (Robb et al., 2018). One of the ways that performing artists may try to manage excessive stress is with alcohol. For example, one large study suggested Australian performing artists may consume alcohol at higher levels than the general population, often in direct relation to managing performance work-related stress (Szabó et al., 2020).

Further to the stressors outlined above, and given that performers experience frequent criticism (Ascenso et al., 2017), performers may lean on self-critical approaches. However, few studies have actively explored this. One study identified how patterns of perfectionism – which typically involves a significant component of self-criticism (Dunkley et al., 2006) – among performing artists contributed to various personal and interpersonal difficulties (Hill et al., 2015). The range of factors described thus far implicate a self-relating style among performing artists that may impact their wellbeing, for example by motivating performance through highly self-critical ways. Finding methods to better relate to oneself may be situated as a key protective mechanism for mental health and wellbeing.

The ability to be self-compassionate may act as a protective buffer against some of the environmental stressors and self-criticism often present for performers (Walton et al., 2022). While many varying definitions for compassion exist (Strauss et al., 2016), here we define compassion as the sensitivity to suffering in self and others, with a commitment to try to alleviate and prevent it (Gilbert, 2014). While compassion can both be given and received to and from others, *self-*compassion (SC) is therefore the expression of compassion toward oneself. There is now a large literature on compassion and SC, spanning much of psychology and related fields. While a thorough review of SC’s mechanisms is beyond the scope here, SC works by reducing the psychological and physiological harms of threat-based cognition and emotion by instead developing soothing responses to distress which allow space for compassionate and growth-focused motivation (Kirby, 2025; Neff, 2023; Gilbert, 2020). Evidence for the benefits of SC have been demonstrated in several ways. Meta-analyses have shown that self-compassion is consistently positively associated with better mental health and wellbeing (e.g., MacBeth and Gumley, 2012; Zessin et al., 2015). In addition, fears of SC are associated with worse outcomes relating to mental health (Kirby et al., 2019). Finally, CBIs which actively aim to improve mental health in both community and clinical populations by increasing SC have proven successful (Millard et al., 2023; Petrocchi et al., 2023). Despite the well-established literature on the benefits of SC and CBIs, very little research has explored how SC can be of value to performing artists.

Prior to exploring the small literature among performing artists, it is worth emphasizing that there is reason to think SC may be beneficial due to the large body of research with athletes in sport. This literature offers insight into the beneficial role of SC in performance environments (Cormier et al., 2023; Röthlin et al., 2019). Among several other positive outcomes among athletes, the ability to be self-compassionate has been associated with better mental health and wellbeing (Walton et al., 2020, 2024), increased prosocial behavior (McEwan and Zhang, 2023; Zhang et al., 2024), less harmful perceptions of body image (Adam et al., 2021; Eke et al., 2019), and the ability to better manage adversity and setbacks (Tremblay et al., 2023; Wilson et al., 2019). Broadly, a self-compassionate approach to performance-related stressors may be helpful by reducing threat-based emotions and cognitions and instead motivating corrective or soothing behavior in the face of distress. Given that many of the stressors that present in sports are applicable to the performing arts (Walton et al., 2022), research with artistic performers is warranted. However, little research exists in the peer-reviewed literature that adequately examines the role of SC on performing artists’ mental health, or the potential for CBIs to target distress in this population.

While this literature in sport holds promise for performing artists, there are important differences between these populations so that specific investigation of SC with performing artists is needed. Among other relevant differences, performing artists navigate artistic and culturally different environments to sport and often receive feedback that is more subjective rather than based on relatively objective markers of success or winning. To date, a small number of studies have explicitly explored SC among performing artists. Kelley and Farley (2019) originally explored SC levels among music students as compared to non-music students, finding no meaningful differences. Within musicians however, these authors later went on to identify a strong negative relationship between SC and performance anxiety among college-level music majors (Farley and Kelley, 2023), suggesting that SC may play a protective role in alleviating key sources of stress among performers. Another study asked undergraduate women students to imagine a hypothetical ballet-based scenario and reported SC to be negatively correlated with state and trait physique anxiety, fear of negative evaluation among other factors (Tarasoff et al., 2017). To our knowledge, no other studies have meaningfully examined the role of SC on mental health among a diverse population of performing artists.

Despite growing work demonstrating the beneficial role of SC on health and performance in sport, the potential for intervention in performing artists remains largely unexplored (Walton et al., 2022). Early work demonstrated that a brief workshop and 7-day compassionate writing intervention led to increased SC, and reduced self-criticism, rumination, and concern over mistakes among 29 varsity women athletes (Mosewich et al., 2013). A much more comprehensive program which adapted the Mindful Self Compassion Program (Neff and Germer, 2013) is RESET (Resilience and Enhancement in Sport, Exercise, & Training; Kuchar et al., 2023), which was explored among a large sample of collegiate athletes. This program took place over six 1-h sessions, and while generally following the Mindful Self-Compassion program content, chose not to make use of the term ‘compassion’ to avoid potential sources of resistance. Findings suggested that those in the intervention with moderate to low SC at baseline, showed significant increases in the construct compared to the control group (Kuchar et al., 2023). While findings such as these are positive for the field of sport, to our knowledge no published interventions for SC exist among performing artists. It is therefore unclear whether established CBIs or those specifically designed for sport are applicable and appropriate to performing artists. Preliminary proof-of-concept research is needed to identify if and how, CBIs can be made relevant and appliable to this population, prior to more comprehensive intervention design and testing.

SC is likely helpful for performing artists. However, there is currently no published research exploring the relationship between SC and mental health, or the potential application of CBIs among performing artists. This two-study program of work therefore fills an important gap by aiming to explore how SC is related to performer mental health, and to develop preliminary evidence as to how a CBI could be developed.

## 2 Study 1 method

### 2.1 Aims and hypotheses

The ability to respond to one’s own distress with compassion has been repeatedly shown to positively influence the severity of psychological distress and mental ill-health. Yet, this relationship has not been explored among performing artists. Thus, study 1 addresses this key gap in the literature by contributing understanding as to the relationship between SC and mental health. Given the limited research on SC among performing artists, most of our expectations were informed through research among athletes and the general population. The primary hypothesis in study 1 was that higher SC would be associated with better mental health (specifically, lower symptoms of depression/anxiety; higher wellbeing) among performing artists when controlling for other key demographic and risk factors that are known to be problematic among performers (stress, body image, and alcohol use).

### 2.2 Participants

Eligibility criteria were for participants to be (i) ≥18 years old and (ii) currently studying or working as a musician, dancer, or actor in Australia. Participants were recruited online (described in procedure). We emphasize upfront that unfortunately, the final dataset was heavily impacted by both missing and fraudulent data entries. A range of strategies were decided post-hoc to remove these participants to ensure only reliable data was used. First, the results from 250 consenting participants were removed as they were based overseas or showed a pattern illustrative of suspicious or fraudulent responses using the Qualtric’s Q_RecaptchaScore (bot detection score based on Google’s invisible reCaptcha technology) as an indicator. Adding suspicion to these removed participants, over 80% of the excluded records were completed on the same date. Second, we chose to include only those participants who had completed at least the demographics section included in the survey, which led to the further exclusion of 102 records. It is acknowledged that this substantial removal of data is far from ideal. This led to a total of 211 participants being included in the final sample which we believe to be reliable.

### 2.3 Procedure

This was a cross-sectional study, with data collected through a Qualtrics survey, hosted online between November 2022 and April 2023. The survey was shared with potential participants primarily via word of mouth and snowballing recruitment procedures. Authors shared with known industry participants as well as via social media (X, LinkedIn). The study was partnered with The Arts Wellbeing Collective (Arts Centre Melbourne), who shared among their networks via newsletters and social media. The study was also advertised with two performing artists websites. This methodology meant it was not possible to determine the response rate. Upon clicking the link to the survey, participants were presented with a detailed Plain Language Statement and Consent Form, prior to completing the survey. At the end of the survey, participants could access a voluntary unlinked survey to go into the running for one of twenty $100 gift vouchers. Study 1 and 2 had combined Institutional Ethics approval (2023–22,353–35,851-4).

### 2.4 Measures

Participants were asked to respond to a range of demographic questions. The measures outlined below were then randomized across participants to reduce order effects. The measures were broadly categorized into the following categories: (i) self-compassion, (ii) mental health, and (iii) risk factors.

Three complementary measures of SC were used. The Self-Compassion Scale (SCS; Neff, 2003) is the most established and widely used scale in SC studies, and thus we felt it important to include to align with established literature. However, concerns relating to the scale exist (e.g., Muris and Otgaar, 2022), and therefore we chose the SC version of the Compassionate Motivation and Action Scale (CMAS-SC; Steindl et al., 2021) as our central measure of SC, given its ability to measure self-compassionate intentions and distress tolerance. While our central analysis focuses on the CMAS, both measures are presented for the reader so that comparisons with existing literature using the more familiar SCS can be made. Finally, we collected fears of SC, given they are known to be common among high-performing athletes, associated with worse mental health, and critical to understand in the context of CBI.

We then included a range of measures broadly reflecting mental health and potential risk factors. Measures of depression and anxiety were selected due to the high prevalence of these as common mental health disorders. We included a measure of wellbeing to capture positive aspects of mental health. Finally, we included risk factors shown in the literature to be prevalent among performing artists in perceived stress, perceptions of body image, and alcohol use. These measures are described in more depth below.

#### 2.4.1 Compassionate Motivation and Action Scale (CMAS-SC)

The CMAS-SC (Steindl et al., 2021) was used as the primary measure of SC. Specifically, the CMAS-SC assesses compassionate motivation and behavior. The scale consists of eighteen items across three factors assessing “Intention” (five items), “Distress Tolerance” (seven items), and “Action” (six items). Participants indicated how much they agreed with the statements, using a 7-point Likert scale from 1 (strongly disagree) to 7 (strongly agree). The total and subscale scores were used, with higher scores indicating higher levels of SC. The Intention subscale describes one’s intention to engage in self-compassionate behaviors, the Distress Tolerance subscale assesses the ability to cope with one’s distressing feelings when approaching one’s own suffering, and the Action subscale describes how an individual has engaged with self-compassionate behavior over the last week. In the current sample, the total score McDonalds Omega was 0.97.

#### 2.4.2 Self-Compassion Scale (SCS)

The 26-item SCS (Neff, 2003) was used as a secondary measure of SC in the current study. Participants responded on a five-point Likert scale ranging from 1 (almost never) to 5 (almost always). A mean total score was calculated (higher scores indicate greater SC). We also examined the two and six-factor subscales, which are split across positive or self-compassionate domains (Self-kindness, Common humanity, Mindfulness) and negative or uncompassionate domains (Self-judgment, Isolation, Over-identification) (see Muris and Petrocchi, 2017). In the current sample, the total score McDonalds Omega was 0.94.

#### 2.4.3 Fears of (self) Compassion Scale (FSCS)

The 15-item ‘For Self’ subscale of the FSCS (Gilbert et al., 2011) was used to examine fears around providing and receiving compassion to self. We used this measure in addition to the SCS and CMAS-SC given evidence that fears of compassion is a key block to intervention work, and is also associated with worse mental health outcomes. Participants responded on a five-point Likert scale ranging from 0 (do not agree at all) to 4 (completely agree) to each statement. Total scores range from 0 to 60 (higher scores indicate more fears to self-compassion). In the current sample, the total score McDonalds Omega was 0.96.

#### 2.4.4 Warwick-Edinburgh Mental Wellbeing Scale (WEMWBS)

The 14-item WEMWBS (Tennant et al., 2007) was used to examine wellbeing. Participants responded on a five-point Likert scale ranging from 1 (none of the time) to 4 (all of the time). Total scores range from 14 to 70 (higher scores indicate higher wellbeing). In the current sample, the total score McDonalds Omega was 0.95.

#### 2.4.5 Patient Health Questionnaire 9 (PHQ9)

The 9-item PHQ9 (Kroenke et al., 2001) was used to examine symptoms of depression. Participants responded to each statement on a four-point Likert scale ranging from 0 (not at all) to 3 (nearly every day). Total scores range from 0 to 27 (higher scores indicate worse depression). In the current sample, the total score McDonalds Omega was 0.94.

#### 2.4.6 Generalized Anxiety Disorder 7 (GAD7)

The 7-item GAD-7 (Spitzer et al., 2006) was used to examine symptoms of general anxiety. Participants responded to each statement on a four-point Likert scale ranging from 0 (not at all) to 3 (nearly every day). Total scores range from 0 to 27 (higher scores indicate worse anxiety). In the current sample, the total score McDonalds Omega was 0.94.

#### 2.4.7 Perceived Stress Scale (PSS)

The 10-item PSS (Cohen et al., 1983) was used to examine perceived stress. This was used as a more concise alternative to assessing exposure to stressors experienced by performers. Participants responded to each statement on a five-point Likert scale ranging from 0 (never) to 4 (very often). Total scores range from 0 to 40 (higher scores indicate worse perceived stress). In the current sample, the total score McDonalds Omega was 0.89.

#### 2.4.8 Body Appreciation Scale-2 (BAS2)

The 10-item BAS2 (Tylka and Wood-Barcalow, 2015) was used to examine body appreciation, as a marker of broader perceptions of body image. Participants responded to each statement on a five-point Likert scale ranging from 1 (never) to 5 (always). Scores are averaged (higher scores indicate better body appreciation). In the current sample, the total score McDonalds Omega was 0.96.

#### 2.4.9 Alcohol Use Disorders Identification Test (AUDIT-C)

The 3-item AUDIT-C (Saunders et al., 1993) was used to examine hazardous drinking. Participants responded to each statement on a five-point scale (each item using different responses). Total scores range from 0 to 12 (higher scores indicate more hazardous alcohol use). In the current sample, the total score McDonalds Omega was 0.86.

### 2.5 Data analysis

All analyses were conducted by a senior biostatistician (CXG) and were not pre-registered. The association between potential SC and risk factors were compared using the Pearson correlation coefficient, to confirm key relationships between self-compassion, risk factors, and mental health outcomes. Linear regression models were chosen as the analytical approach as they allow comparison regarding the strength of associations between SC and risk factors (SC intention and distress tolerance, alcohol use, body appreciation, and perceived stress) on the three primary mental health outcomes (anxiety, depression, and wellbeing). The intention of this analysis was to determine the impact of SC on mental health and wellbeing over and above these risk factors and control variables. The CMAS Behavior subscale was not used given its measurement reflects change over the last week. For each outcome, two sets of models were conducted. The first set included a partially adjusted model with individual factors included in separate regression models controlling for confounding factors of age, gender identity, sexual orientation, role (performers vs. students), and performer type. Then in the fully adjusted model, all factors were included jointly with confounding factors. SC and risk factor variables were standardized to allow easy comparison between risk factors. All analyses were repeated for crude outcome scores and standardized outcome scores. Missing data were addressed using Multiple Imputation by Chained Equations (MICE) with 20 imputed datasets. Results were pooled using Rubin’s rule (Rubin, 1987). All analysis were conducted using R version 4.4.1 (2024/06/14).

## 3 Study 1 results

**Figure 1:**
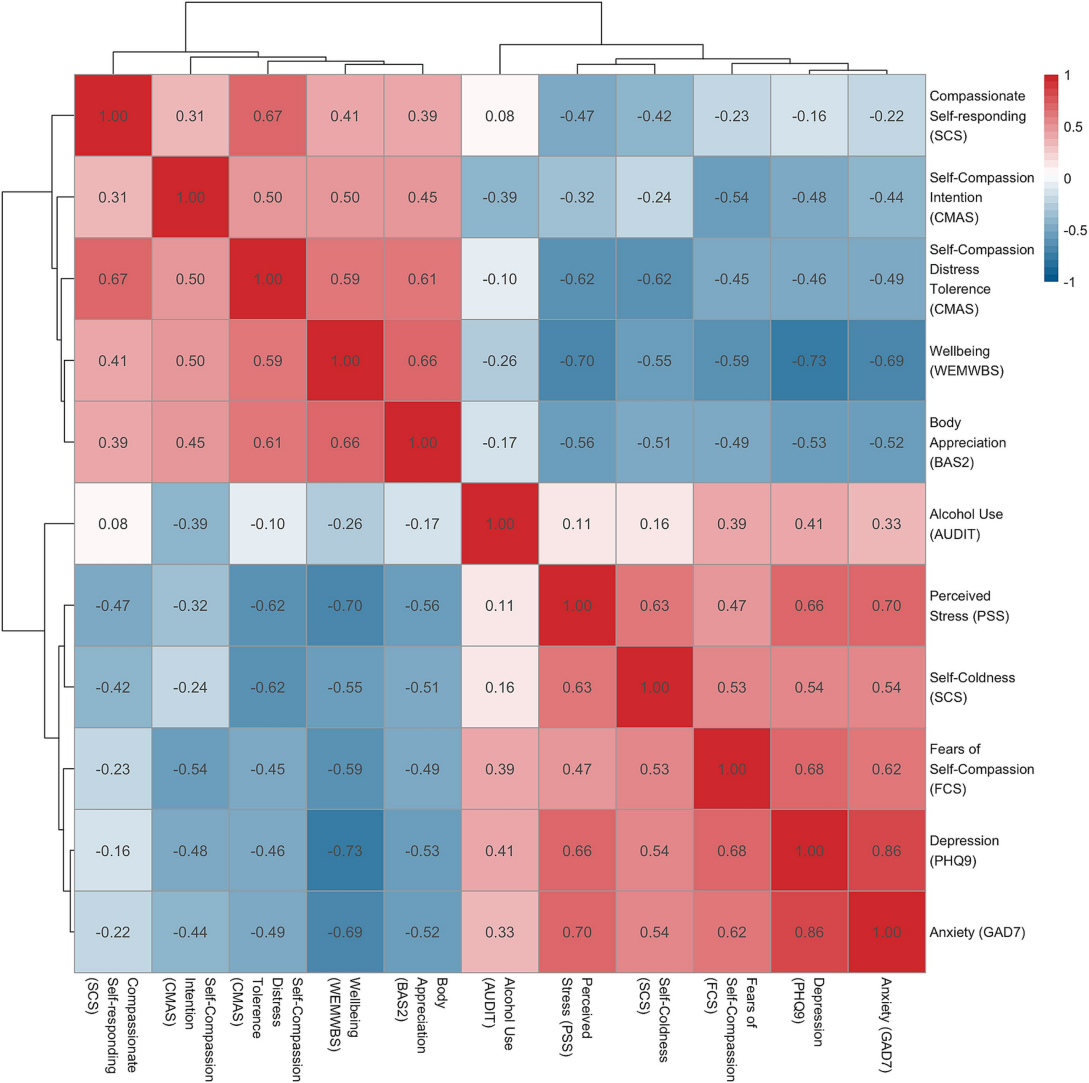
Correlations for the variables used in the study.

Key descriptives for the sample are presented in Table 1. Over half the sample (56%) reported at least one current or past mental health diagnosis. Supporting this, 51% of GAD7 scores and 52% of PHQ9 scores fell above cut-off scores for likely anxiety or depression, respectively. Further detail on mental health and self-compassion split by performer type are included in Supplementary Tables 1, 2, which demonstrated that dancers reported significantly higher rates of stress, anxiety, depression, and fears of self-compassion, along with lower rates of wellbeing and SC. Figure 1 provides correlations between the key variables, with all demonstrating relationships in line with broader literature. Primarily, the various measures of SC were negatively associated with depression, anxiety, stress, and alcohol use, while positively associated with wellbeing and body appreciation. Fears of self-compassion were more strongly correlated with depression and anxiety than measures of SC. The CMAS intention and distress tolerance subscales showed larger correlations with mental health than the compassionate responding items of the SCS.

**Table 1:** Participant descriptives.

	Overall (*N* = 211)
Age*	32.7 (13.4)
Gender	
Women	131 (62.1%)
Men	62 (29.4%)
Non-Binary or another descriptor	18 (8.5%)
Sexual orientation	
Heterosexual	132 (62.6%)
Other	79 (37.4%)
Aboriginal or Torres Strait Islander	
No	203 (96.2%)
Yes	8 (3.8%)
Role	
Performer	157 (74.4%)
Student	54 (25.6%)
Performer Type	
Music	111 (52.6%)
Dance	68 (32.2%)
Acting	32 (15.2%)
Self-reported Mental Health Diagnosis (ever)	
Yes	117 (55.5%)
No	73 (34.6%)
Other	21 (10.0%)

*One participant did not report their age.

**Figure 2:**
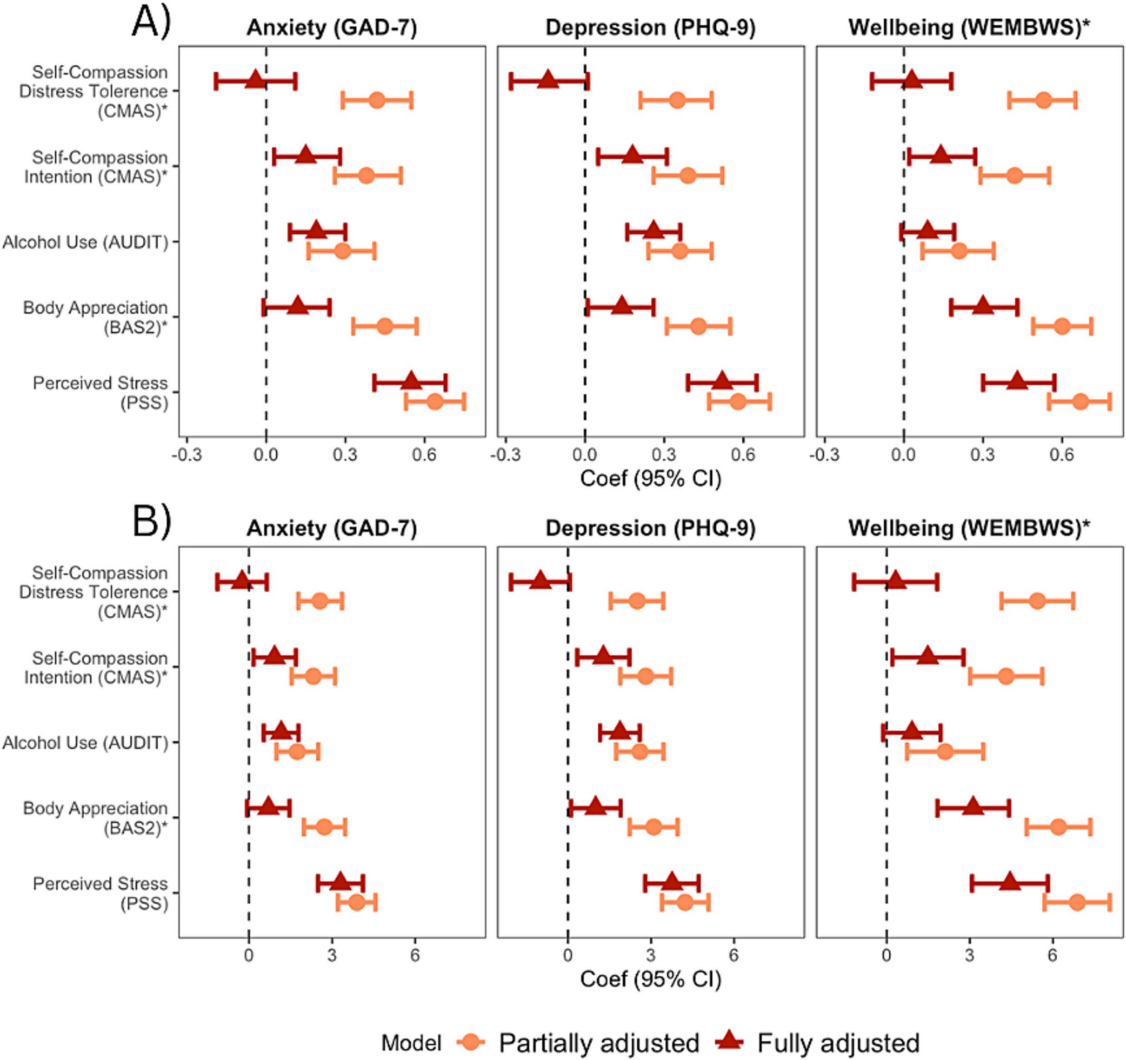
Results from partially and fully adjusted linear regression models for three outcomes. **(A)** with outcome standardized and **(B)** with outcome unstandardized. Missing data were imputed and all risk factors were standardized; * direction reversed for ease of interpretation.

Next, a series of regressions were conducted to examine the effect of SC on depression, anxiety, and wellbeing, while controlling for relevant factors. The CMAS had stronger and more consistent correlations with mental health than the self-compassionate subscale of the SCS, and was used as the primary measure of SC. Standardized and unstandardized findings with imputed data are shown in Figure 2 with greater detail in Supplementary Table 3. Partially adjusted regressions controlling for age, gender identity, sexual orientation, role (performers vs. students), and performer type showed that SC-intention and SC-distress tolerance, along with stress, body appreciation, and alcohol use were all significantly associated with anxiety, depression, and wellbeing.

Regarding depression in the fully adjusted model, a one SD increase in perceived stress was associated with a 0.52-SD increase in PHQ9 scores (*p* < 0.001), one SD increase in alcohol use was associated with a 0.26-SD increase (*p* < 0.001), a one SD increase in SC-intention resulted in a 0.18-SD decrease (*p* = 0.009), and a one SD decrease in body appreciation resulted in a 0.14-SD decrease (*p* = 0.031). For anxiety, a one SD increase in perceived stress was associated with a 0.55-SD increase in GAD7 scores (*p* < 0.001), a one SD increase in alcohol use led to a significant 0.19-SD increase (*p* < 0.001), and a one SD increase in SC-intention resulted in a 0.15-point decrease (*p* = 0.019), while neither body appreciation nor SC-distress tolerance were significantly associated with GAD7 scores. Finally, considering wellbeing, a one SD increase in perceived stress was associated with a 0.43-point decrease in WEMWBS scores (*p* < 0.001), a one SD increase in body appreciation resulted in a 0.30-SD increase (*p* = 0.001), and a one SD increase in SC-intention resulted in a 0.14-SD increase (*p* = 0.026). Overall, perceived stress and SC-intention had the most consistent and largest effects on mental health among performing artists.

## 4 Study 2 methods

### 4.1 Aims and hypotheses

Having found that SC was meaningfully associated with mental health and wellbeing among performing artists, we set out to examine how a CBI could assist performing artists. Given that limited research exists exploring SC among this population, we aimed to examine the proof of concept for a CBI among performing artists using a brief intervention. We were interested in examining the acceptability, feasibility and potential effectiveness of a brief CBI. Given the early stages of this research, we chose to integrate qualitative feedback from participants to understand their perceptions toward such an intervention. Mixed methods have been promoted as helping researchers to understand “not only whether a novel intervention works, but also how and why, or why not” (Fetters and Molina-Azorin, 2020). No specific hypotheses are provided as the study is exploratory. The descriptive data is provided to give preliminary estimates of acceptability, feasibility, and effectiveness, and supplement the qualitative data. The study aims to address the following research questions: (1) how might SC support performing artists, and (2) what are participants’ perspectives about further development of CBIs, following their use here?

### 4.2 Stage of development: Proof of concept

This was a non-randomized single group study aimed at establishing proof of concept for CBIs among performing artists. Our broader objectives are to develop a comprehensive CBI for performing artists to be subsequently tested with pilot and then RCT methodology. However, no literature exists on CBIs in the performing arts, and therefore the methodology employed was designed to provide preliminary insights to the concept of developing a more comprehensive CBI for this population and initial signs of potential efficacy.

### 4.3 Procedure

Participants were recruited using the same method as Study 1. Some participants provided their interest and contact details for Study 2 at the completion of Study 1. Interested individuals signed up to attend a 90-min online workshop delivered by the study team. The workshop introduced the concept of SC and its potential relevance to performing artists and provided a summary of the proposed study. At the end of the workshop, participants were invited to register for the study through a Qualtrics link which provided them with a Plain Language Statement, Consent Form, and a brief battery of assessments. Following completion of the pre-intervention questionnaires, participants were provided with a link to access mp3 files for the compassion meditations. Participants were emailed an online weekly diary at the end of weeks 1, 2, and 3 to keep a record of participation. Participants could also opt in to receive text reminders to use the meditation every 3 days. At the end of the 3 weeks, participants were emailed the follow-up questionnaire and were offered the opportunity to take part in a brief semi-structured interview about their experiences. Participants were reimbursed AUD$50 for completion of the study, and AUD$50 for completion of the interview.

### 4.4 Intervention

The intervention took the form of a workshop and continued use of three guided meditations. The workshop was held online via zoom and led by authors CW and JK. The goal of the workshop was to introduce participants to the construct of SC, address common misconceptions or fears, and emphasize its role specifically to performance. The meditations were scripted and recorded by author JK, who is an internationally recognized expert in Compassion Focused Therapy (Kirby, 2025; Petrocchi et al., 2024). The recordings were all based on established and well recognized exercises featured within Compassion Focused Therapy (Petrocchi et al., 2024). However, to enhance the relevance to participants, the wording was slightly adjusted to be more specific to performing artists. Both the workshop and exercise scripts are freely available at https://osf.io/3kdsg/. These exercises were titled (1) Compassionate Practice for Self and Others, (2) Compassionate Self for Anxiety, and (3) Compassionate Self for Self-Criticism. Each was approximately 10 min in length. Participants were instructed to try and use one meditation each day over the three-week period (i.e., ~21 times), while acknowledging this may not have always been possible. Participants were encouraged to use the exercises when it made sense to them, and was not mandated for specific instances (e.g., before or after a performance). The intervention period was chosen to expand upon previous work using a similar methodology, which found 1 week to be too brief (Kim et al., 2020).

### 4.5 Participants

Eligibility criteria matched Study 1. We aimed to recruit 30 participants for this study aligned with general rule of thumb suggestions for pilot or similar studies. A total of 33 participants consented to take part in Study 2 after completion of the workshop. Unfortunately, 17 of these were subsequently geolocated to Nigeria and categorized as fraudulent given the inclusion criteria explicitly required current Australian residence in addition to suspicious behavior from participants (repeated and unusual emails). Two others did not complete any item from the baseline survey after consenting, and four did not complete the intervention or follow up. Given the exploratory nature of this study, all were deleted. This left ten eligible participants who completed all parts of the intervention. Eight of these participants elected to participate in an interview (five women, three men; mean age: 45).

### 4.6 Measures

Participants completed a range of demographic questions and established questionnaires already described in Study 1 Methods. Three scales were used to assess SC (SCS; CMAS; FSCS), two scales were used to assess mental health and wellbeing (WEMWBS; Depression, Anxiety and Stress Scales), and the BAS-2 was used to assess body appreciation. Finally, three scales to assess intervention acceptability, appropriateness, and feasibility were used at follow-up. The new scales used in Study 2 are described below.

#### 4.6.1 Depression, Anxiety and Stress Scales (DASS-21)

The DASS-21 (Lovibond and Lovibond, 1995) was used to examine psychological distress broadly. Participants responded to each statement on a four-point Likert scale ranging from 0 (did not apply to me at all) to 3 (applied to me very much or most of the time). The scale consists of three subscales measuring depression, anxiety, and stress. Total scores were calculated by summing all items (higher scores indicate worse distress).

#### 4.6.2 Implementation Outcome Measures

The 4-item Acceptability of Intervention Measure (AIM), 4-item Intervention Appropriateness Measure (IAM), and the 4-item Feasibility of Intervention Measure (FIM) (Weiner et al., 2017) were used to examine participant perspectives of acceptability, appropriateness, and feasibility. Participants responded on a five-point Likert scale ranging from 1 (completely disagree) to 5 (completely agree) to each statement about the intervention. Mean scores are reported, with higher scores indicating more positive perspectives about the intervention.

#### 4.6.3 Bespoke questions to clarify proof of concept

Seven additional items were provided which capture participant perspectives on the intervention and future research interest. These aimed to capture participants key learnings (understanding of SC), reflections on SC (enjoyment, perceived improvements), and potential needs for future intervention (tailoring, detail). Example items include “After this project I feel like I better understand what self-compassion is” and “In general, it would be helpful for performing artists to learn how to be more self-compassionate.”

### 4.7 Data analysis

This was a mixed-methods evaluation study, which included triangulating quantitative descriptive data with qualitative evaluation via semi-structured interviews. Due to the sample size and nature of this study, only descriptive and summary statistics are provided for the quantitative data. Qualitative data was analyzed through reflexive thematic analysis (Braun and Clarke, 2019; Clarke and Braun, 2021), following the six phases outlined by these authors. First, CW familiarized himself with the data by reading each transcript multiple times. This process was strengthened by CW conducting all interviews, rewatching recordings, and editing the transcripts. During this phase, CW made general notes on initial thoughts and observations. Next, he systematically labeled and coded key ideas, aligning them with the initial research aims relating to acceptability, feasibility and potential effectiveness of CBIs. This process included producing both semantic and latent codes. In the third phase, CW generated initial themes by grouping codes into broader patterns of meaning, using mind maps to organize and experiment with these candidate themes. In the fourth phase, he refined the themes further by asking reflective questions, as suggested by Clarke and Braun (2021), and discussing theme progression on multiple occasions with critical friends (JK and MO). In the fifth phase, CW confirmed the naming that best captured the themes overarching meanings. During this phase, participant quotes were incorporated within theme names to emphasize participant voice (Clarke and Braun, 2021). Finally, CW wrote the narrative of the results, incorporating under these themes the key ideas and illustrative quotes, which were then reviewed and refined by all authors as well as during peer review. All analysis was conducted using NVivo. No participant knew the interviewer prior to participation. The qualitative results are provided following the quantitative findings; however, we do not privilege one form of evidence as more or less important. Both forms are seen as complementary to understanding participant experiences of using the intervention.

### 4.8 Reflexivity and philosophical positionality

A pragmatic research paradigm was adopted, in that the authors accept that there are singular and multiple realities but that this preliminary work is focused toward solving ‘practical problems’ in the ‘real world’ (Gibson, 2016; Yvonne Feilzer, 2010). All interviews and the qualitative analysis were conducted by author CW (He/Him); a psychologist with both research and applied expertise in clinical sport psychology and compassion focused therapy. CW has no lived experience in the performing arts but has previously completed a provisional psychologist placement within a dance school and worked with performing artists experiencing mental ill-health in his clinical practice. Throughout the interviews and analysis, CW remained cognizant of how his academic interests in the positive role of CBIs could affect his interpretation of participant perspectives in favorable ways.

### 4.9 Conduct of interviews

Having completed the follow up survey, participants could opt in to an interview. Interviews were conducted by author CW on zoom. Participants gave informed verbal consent to take part in the interviews. Participants were asked pre-defined questions which covered their perspectives and understanding of SC and its relationship to mental health and performance, perceived changes following the intervention, likes and dislikes of the intervention, and suggestions to make a larger intervention most attractive to performing artists. Interviews were recorded using Zoom’s in-built recording option, and mp3 files were transcribed by an external transcription service, REV. Participants did not view or provide feedback on these transcripts.

## 5 Study 2 results

### 5.1 Quantitative results

**Figure 3:**
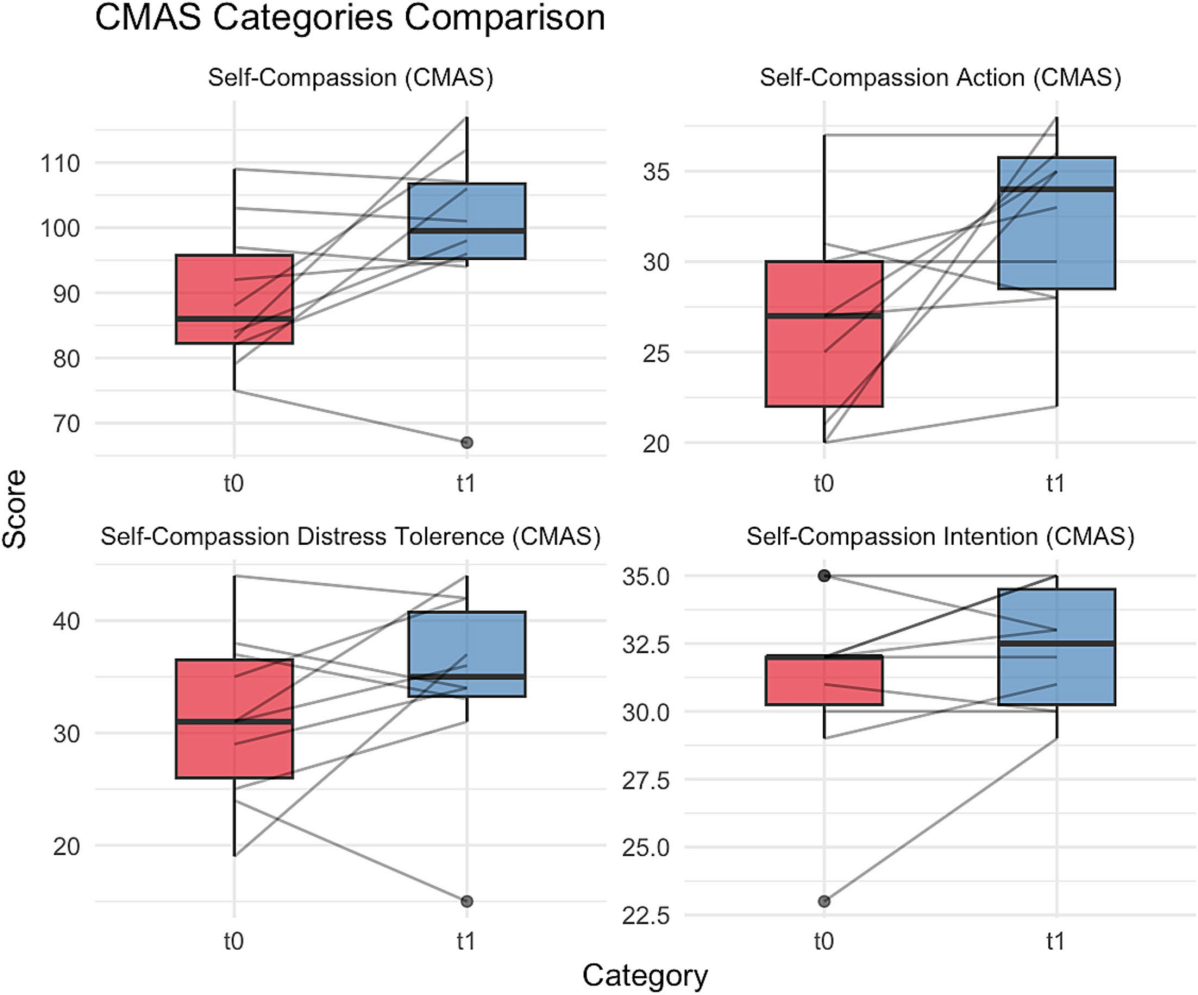
Changes in CMAS total and subscale scores from baseline (t0) to follow up (t1).

The ten participants had a mean age of 43.5y (SD = 12.6) and were made up of six women (60%), three men (30%), and one non-binary individual (10%). Seven participants identified as heterosexual (70%). The participants identified themselves as primarily working in music (*n* = 5; 50%), acting (*n* = 3; 30%), and dance (*n* = 2; 20%), with half of the participants identifying as multidisciplinary. Nine participants derived income from performing, with one participant a performing artist who was neither a student nor currently deriving an income from performing. Mean difference and effect size measures in Table 2 suggested that overall, participants self-reported higher rates of SC (SCS *d* = 0.74; CMAS *d* = 0.73), body appreciation (*d* = 0.34) and wellbeing (*d* = 0.25), while reporting lower rates of fears of SC (*d* = −0.40) and psychological distress (DASS-21 *d* = −0.51), following the intervention. Figure 3 shows these key pre-post changes in the CMAS. Further corresponding to these results, 9 of the 10 participants agreed they now better understood self-compassion, and 8 out of the 10 agreed they had become more self-compassionate. Table 3 provides level of agreement relating to acceptability and feasibility. Mean scores for the AIM, IAM, and FIM suggested good acceptability, appropriateness, and feasibility (all >4/5). Further, level of agreement to bespoke questions about the intervention showed that every participant agreed that performing artists should learn about SC and were interested in a future larger intervention. According to self-reported diaries, participants used the exercises on approximately 14 of the 21 days, suggesting relatively good uptake.

**Table 2:** Pre-post descriptives for outcomes relating to self-compassion and mental health.

Measures	Pre	Post	Difference (95% CI)	Effect size D (95% CI)
CMAS Total	89.20 (10.93)	99.30 (13.68)	10.1 (20.6, −0.4)	0.73 (1.45, −0.02)
CMAS Intention	31.10 (3.41)	32.30 (2.26)	1.2 (2.9, −0.5)	0.54 (1.22, −0.17)
CMAS Distress Tolerance	31.30 (7.47)	34.80 (8.20)	3.5 (9.4, −2.4)	0.44 (1.12, −0.25)
CMAS Action	26.80 (5.49)	32.20 (5.07)	5.4 (10.4, 0.4)	0.82 (1.55, 0.05)
SCS Total	2.95 (0.68)	3.25 (0.73)	0.3 (0.6, 0.0)	0.74 (1.46, −0.01)
SCS Self-Kindness	3.12 (0.85)	3.46 (0.92)	0.3 (0.6, 0.0)	0.83 (1.57, 0.06)
SCS Self-Judgment	3.44 (0.92)	3.10 (1.11)	−0.3 (0.1, −0.8)	−0.62 (0.10, −1.32)
SCS Common Humanity	3.55 (0.89)	3.52 (0.80)	0.0 (0.4, −0.5)	−0.04 (0.61, −0.69)
SCS Isolation	3.50 (1.07)	2.72 (1.03)	−0.8 (−0.3, −1.3)	−1.12 (−0.27, −1.93)
SCS Mindfulness	3.62 (0.71)	3.65 (0.70)	0.0 (0.4, −0.4)	0.05 (0.70, −0.61)
SCS Over-Identification	3.60 (0.78)	3.28 (0.94)	−0.3 (0.3, −1.0)	−0.38 (0.31, −1.04)
SCS Compassionate Self-responding	3.43 (0.64)	3.54 (0.63)	0.1 (0.4, −0.2)	0.32 (0.98, −0.36)
SCS Uncompassionate Self-responding	3.51 (0.86)	3.03 (0.88)	−0.5 (−0.1, −0.9)	−0.86 (−0.08, −1.61)
Fears of Self-Compassion	21.10 (16.30)	18.40 (14.09)	−2.7 (2.4, −7.8)	−0.40 (0.29, −1.07)
WEMWBS Total	44.80 (6.05)	46.40 (7.73)	1.6 (6.4, −3.2)	0.25 (0.91, −0.42)
DASS-21 Stress	9.60 (5.25)	7.90 (5.38)	−1.7 (0.5, −3.9)	−0.58 (0.14, −1.27)
DASS-21 Anxiety	3.50 (2.76)	3.60 (4.43)	0.1 (2.1, −1.9)	0.04 (0.69, −0.62)
DASS-21 Depression	5.50 (2.99)	4.00 (3.06)	−1.5 (1.0, −4.0)	−0.46 (0.24, −1.13)
DASS-21 Total	18.60 (9.12)	15.50 (11.76)	−3.1 (1.5, −7.7)	−0.51 (0.19, −1.19)
BAS2 Total	33.70 (5.42)	35.10 (6.71)	1.4 (4.5, −1.7)	0.34 (1.00, −0.34)

CMAS=Compassionate Motivation and Action Scales; SCS=Self-Compassion Scale; WEMWBS = Warwick-Edinburgh Mental Wellbeing Scale; DASS-21 = 21-item Depression, Anxiety, and Stress Scale; BAS2 = Body Appreciation Scale-2.

**Table 3:** Measures of acceptability and feasibility.

Measure	Median (min, max)	Disagree	Neither agree nor disagree	Agree	Strongly agree
Meditation sessions completed^1^	13.5 (7, 21)				
Intervention Acceptability	4.0 (3.5, 5)				
Intervention Appropriateness	4.0 (3.25, 5)				
Intervention Feasibility	4.0 (3.25, 5)				
Better understand self-compassion	4.0 (3, 5)	0 (0%)	1 (10%)	6 (60%)	3 (30%)
Improved self-compassion	4.0 (3, 5)	0 (0%)	2 (20%)	6 (60%)	2 (20%)
Enjoyed working on self-compassion	4.0 (3, 5)	0 (0%)	1 (10%)	6 (60%)	3 (30%)
PAs should learn self-compassion	5.0 (4, 5)	0 (0%)	0 (0%)	4 (40%)	6 (60%)
Interest in bigger intervention	5.0 (4, 5)	0 (0%)	0 (0%)	3 (30%)	7 (70%)
Would recommend intervention	5.0 (3, 5)	0 (0%)	1 (10%)	3 (30%)	6 (60%)
Should be tailored for PAs	4.5 (2, 5)	1 (10%)	2 (20%)	2 (20%)	5 (50%)

^1One participant did not complete the week 1 diary, and it cannot be reliably determined how many meditations they used that week.^

### 5.2 Qualitative results

Across the 8 interviews, the average length was 48 min (min: 38 m, max: 61 m). Four primary themes were captured which added clarity to the primary research questions. The first theme explores why self-compassion is beneficial to performers’ management of the many internal and professional demands they faced. The second theme overviews some of the fears, blocks, and resistance to SC that came up among performers, especially early in the intervention. The third theme features participants reflections on growth that stemmed from the intervention. Finally, the fourth theme describes key desires and suggestions for future CBI’s among performing artists. Note that all names are pseudonyms.

#### 5.2.1 Theme 1 - “You actually need to do this if you are going to work in this world”: Self-compassion supports performers’ health and performance

Participants were clear that SC was helpful to performing artists. Performers described many of the relevant stressors inherent to their field in the context that SC could be the “antidote.”

##### 5.2.1.1 Responding to internal triggers: self-criticism, perfectionism, and emotional extremes

Performers were broadly described by participants as being susceptible to forms of self-relating that made them vulnerable to stress, burnout, performance decline, and eventually, mental ill-health. As described by Ethan, “*I think it’s probably very common with artists, is that sort of perfectionism… we are our own worst critics…we are less forgiving with our shortfalls than other people might be.*”

Performers described themselves and their peers as particularly self-critical, and this was seen as inherent to performing environments but only effective to a point, before becoming detrimental: “*People who have got destructive inner narratives or who are always looking for the thing they missed or the thing they did not do well are constantly eroding any sense of success, achievement, identity, stability. It’s always about the thing that was missing. And the frightening thing about that is it’s actually quite effective for a lot of people. Unfortunately, it’s not sustainable.*” (Emily).

Ava described that a central focus on finding fault can lead to criticizing oneself, rather than performance: “*We’re often, from a very early age, practicing by ourselves, criticizing what we do, identifying mistakes, trying to fix them. But that critical process can slip really easily into criticizing self, rather than criticizing technique.*”

Sophia put this down to creatives as being “*different*” in some way: “*I think our creative mind just churns more than most maybe.*” She felt that “*the majority of performing artists would be pretty critical and hard on themselves… I think we are all quite similar in the way we approach things. I think it’s sort of an artistic mind type problem.*” She suggested that a disproportionately high number of performers experience mental ill-health because of this: “*a lot of my friends in performing art areas… a lot of them have suffered from depression*.”

A unique perspective provided by Emily was that performing artists must manufacture emotional distress in their performance. She described “*the process of telling stories with emotions is potentially destabilizing for a person because they are going to emotional extremes quite often*.” She gave an example of her friend, “*a woman who was playing a wife in an abusive relationship. She said it was terrible for her mental health because each night, she had to be anxious and afraid.*”

##### 5.2.1.2 The performing arts industry as uniquely problematic

Performers described working within a high-risk environment, in which external stressors are placed upon them. William described that being a performer was “*a bit like throwing yourself at something really scary and potentially very, very painful and humiliating.*” Environmental stressors included things like bullying, financial insecurity, judgment, rejection, and not having structural support systems in place. Ava described that “*the stakes are often really high and really public, and that can be really, really difficult to manage.*”

Judgment was perhaps the most prominent of these stressors and was seen as exacerbating self-criticism: “*you are being given critical feedback every single day, every single second of your performance, as well as to yourself*” (Isabella). This criticism was so difficult because “*the feedback you get is literally about you as a person” (Isabella)*, and *“it can feel so personal all of the time*” (Olivia). Emily described that “*of course people get rejected. They start thinking, what’s wrong with me?*”

Another major stressor was financial stress. William described “*for all of its glitz and glamour, there are hundreds and thousands of performers out there. It’s really hard to make a living out of it. It’s got a lot of glamour, but not a lot of prestige. Almost all performers are fairly poor*.”

Performers’ workplaces were portrayed as often lacking support. Further, free-lance performers may not have a workplace to offer such support at all. As described by Isabella, “*we do not have HR… we do not celebrate ‘are you okay day’ … We do not have access to [Employee Assistance Programs].*”

When describing working for a leading national music-based organization [masked by authors], she described that “*I wasn’t treated like an employee. I was treated like, ‘You’re lucky to be here.’ And these are our professional companies… You’re a freelancer in this environment where you do not get any workplace support.*”

While many comparisons have been made between performers and athletes, Isabella described that performing artists often have much less support: “*As a performing artist, often we are alone in our craft, and we are like athletes. We’re like Olympic athletes. And Olympians have a whole coaching team behind them… As performing artists you do not necessarily have that*.”

##### 5.2.1.3 Self-compassion is well suited to the challenges faced by performing artists

Considering the above difficulties, participants described why SC was beneficial: “*I think self-compassion for performing artists is vital, because it’s an extremely difficult, emotionally difficult industry… If you do not have a way to self-preserve yourself in that environment, and compassion is a really huge aspect, I think, a way of surviving that environment, it’s almost, it’s quite dangerous. You’ve got to find tools to help you get through*” (Isabella).

Performers viewed SC as supporting performance as well as mental health. This related to overcoming hurdles, disappointments, or taking risks. Ava described that SC was “*certainly one of the things that I turn to in terms of dealing with moments where things do not go quite as you would’ve liked them to… because if you are striving at things and if you are putting yourself on the line, then there will be those moments. There’s no way around that unless you want to not challenge yourself… if you are putting yourself out there as a performer, there’ll be those moments where it feels hard*.”

William described SC as a “*helping hand*,” in which: “*To have that capacity to check in very quickly with yourself and say, ‘It’s okay. You’ve put your heart and soul into this and you are giving it your best, and in fact it will be okay.’ It is that reassuring that comes from choosing to like yourself despite the fact that at that moment you are finding it difficult*.”

Returning to Emily’s comments about emotional extremes, she describes how SC might help: “*I think this idea that performers are playing with the physical and emotional expression of emotion, so you can see how important it is to return to a baseline after you have done all this stuff… They’re working within the field of emotion and so there’s almost a need for them to sit quietly afterwards, let their central nervous system equalize again… The idea I’m trying to express is even if they are performing it, even if they are pretending, they are getting this nervous response [that] something really bad is about to happen, you need to actually take care. And I think that there’s a toll that comes with that. It seems like there’s going to be a price that’s paid by the body on that one. And I think a lot of the Buddhist practices around self-compassion and meditation have been about calming the body and the mind at the same time*.”

#### 5.2.2 Theme 2 - “Am I just going to start cramming my ass to the sofa and singing kumbaya?”: Fears, blocks, and resistances to self-compassion

Despite the value of SC identified by participants, there were many blocks that got in the way. Smaller barriers included finding time and energy. More significant fears related to fears around dropping standards or misunderstanding what SC is.

##### 5.2.2.1 The fear that self-compassion might reduce standards

The idea that being self-compassionate is at odds with the high-performance demands of performing artists was frequently raised. Ethan described “*there is that kind of in-built judge in me and the idea of letting yourself off the hook will make you perhaps a less accurate performer or disciplined performer.*” Emily worried that “*If I start tinkering with my psychology, how is that going to affect any kind of performance outcome? Am I just going to start cramming my ass to the sofa and singing kumbaya?*”

Olivia described her thoughts while responding to items in the Fears of Compassion Scale: “*I was like, a hundred percent, yeah, I definitely feel that.*” Interestingly, she went on to describe that she knew this wasn’t accurate on a cognitive level, she still felt it: “*I think this is where theory and… actual practice disconnect for me in quite a significant way. I understand this is not right or correct, but still it feels correct. So, it feels like if I was to be just endlessly self-compassionate, that feels very passive, it feels very complacent*.”

Alexander described his hesitancies at the start of the study: “*the viewpoint that I had of compassion was, ‘oh, well, if you are not feeling it, just do not go practice or do not go to rehearsal’ or those kind of compromising phrases.*” He described initial confusion about the project’s approach: “*How could they be thinking about compassion in this particular way for high achievers? It does not make sense to me.*”

##### 5.2.2.2 Confusion about how to be self-compassionate

These fears reveal a common misunderstanding about what SC is, and many participants described some initial confusion about the practice of SC. Specifically a lot of participants had trouble in identifying how to be self-compassionate: “*I’ve felt I’ve always struggled with self-compassion. And I think personally, to a certain extent… the actual practice of doing it or understanding it*” (Ethan).

“*I feel that a lot of people do not really understand what does that look like? What does self-compassion look like? And it might be different for different people, but… with the meditation specifically, sometimes I would just go, ‘Am I doing this right?’ And just often it would just come up, ‘what is self-compassion?’*” (Alexander).

The discrepancy between theory and practice was further described by Olivia, who had a lot of difficulty being self-compassionate even when she felt it was important: “*I think theoretically I’m like, ‘Yeah, 100%, I’m so bought into self-compassion…’ And then when it actually gets time to apply it to yourself, it’s so much harder to do… Theoretically I can absolutely see and imagine that it would have tremendous impacts on improving mental health and wellbeing. I would recommend it to everybody. I would go out, I’ll tell my parents, I’ll tell my family, tell my friends, tell everybody, ‘Go do the self-compassion thing.’ But when it comes to actually sitting down and doing it for yourself, I think that can be quite challenging*.”

#### 5.2.3 Theme 3 - “I’ve been a lot happier and more positive since I’ve been taking part”: Perceived improvements following the intervention

Supporting the descriptive results presented above, each participant referenced benefits following the intervention, and most appeared to have enjoyed the intervention: “*I really enjoyed the process, having the meditations, it was just enjoyable to do. I really liked it*” (Sophia).

These improvements included perceptions of better handling stressors, improved understanding of SC or themselves, reduced stress, reduced self-blame, and increased positive emotional states. Critically, this was all within the context of maintaining standards: “*I do not think the standard of what I was presenting had dropped*” (Ethan).

Improvements were often an effect of thinking about SC more intentionally, as much as using the meditations. For example, Alexander suggested “*I think that the meditations helped so little in comparison to the amount of time that I just spent thinking about the study.*”

Further, participants could lean on the learned content of the meditations without the recording: “*What I did sometimes was a micro version of it in my head… It gave me words to use to myself. When I was feeling a bit shit, I would say some of the words that was in that meditation to myself*” (Isabella). One participant even described replacing their ritualistic pre-show cigarette with an internal meditation: “*I have used this, not actually switched on the tape, but just used my memory of the tape and my memory of that place that I go to and sat down quiet and did a self-compassionate meditation instead of having a smoke*” (William).

Evidencing self-perceived benefits, several participants described that they had shared the meditations with non-participants. For example, Emily described sharing with her fellow performers before a show: “*I said to [other performer], ‘can you email the cast and send them a link and just say’, ‘Look, I’ve got some homework for you all.’ And saying, ‘Put it in your own words, but we have been taking part in this pilot study. We found it really helpful. We’d really love you to find 10 min this afternoon before our show just to pick one of these meditations and do that*.”

Sophia described that her “*daughter listened to [the Compassionate Self for Anxiety meditation] at some point too, and she enjoyed it too. She’s only young, but yeah, I let her listen to that one.*”

William described “*handing out compassionate performer tapes to a few people over the last couple of weeks*,” including to a performer who had a particularly bad show and was very distressed afterwards: “*I thought to ring her and she was a mess, and she says, “I’m never going on stage again. I’ve been crying all night. I stuffed it up for everybody.” And I talked her down and I sent her a link to your meditations, and she listened to them, and yeah, I’ve kind of enrolled somebody new into the study that you do not even know, and she got right back on that horse.*”

##### 5.2.3.1 Improved self-compassion and mental health

Perceived improvements tended to span performance, wellbeing, and SC. Often, this was noticed in response to stressors, including outside of performance contexts, as described by Emily: “*There’s been some stuff happening. And so whenever we have had a difficult day, we have been using these meditations just as a grounding and a connecting thing because the emotional overload has not come from performance per se. It’s come from just dealing with people who are very unwell. So it feels the same. It’s just originating from a different source. So yeah, they have been really, really helpful and they are continuing as a practice that we are doing*.”

Participants reported improved wellbeing. Ethan described that “*it’s definitely had a significant impact upon my mental wellbeing.*” Sophia stated, “*I’ve felt I’ve been a lot happier and more positive since I’ve been taking part in it too, and more confident*.”

Much of this came down to reduced self-critical tendencies – “*I think it has definitely helped me be a lot more positive towards myself, not be so judgmental*” (Sophia). Ethan described: “*I do, in reflection, feel that I have not been as self-critical during this period.*”

##### 5.2.3.2 Improved understanding of self-compassion

Views about SC, or how one related to it, were described as improving through the study. For example, Olivia, who had previous experience with meditation and SC from a different perspective, described how “*I practice meditation myself, and that’s the Buddhist tradition. So, I suppose my understanding of self-compassion has been quite influenced by that. I feel like maybe I got a slightly different flavor of what self-compassion is from this experience*.”

Isabella described how she came to understand more about her relationship to self-compassion. She described “*I found it a lot easier to be compassionate towards other people, like people I even dislike, then to myself. And I’m very aware of why that is the case, but again, I did not realize there was a discrepancy there*.”

Alexander - who described a lot of blocks to SC at the beginning of the study - described how “*I remember my internal dialogue of the question a lot was, ‘Try to imagine what it would be like if you were a compassionate person to yourself’ and I was, ‘Well, I’d be shit at [my instrument].’ There was a direct argument to that and there was no working that out until I worked it out, that I could not meditate my way through. My journey to self-compassion needed to be filled with violence and strife rather than calm, peaceful sitting. And once I got that, it was a lot easier*.”

#### 5.2.4 Theme 4 - “Across the arts, this intervention would be incredibly useful”: Adapting and implementing compassion-based interventions for performing artists

It was clear that CBIs were valued, with Sophia describing them as “*definitely something performing artists need.*” Participants gave significant specific feedback about their experience using the meditations, and how CBIs could be used going forward. The most broadly relevant views are grouped below as those which can inform future intervention development.

##### 5.2.4.1 Interventions for performing arts can be cross-disciplinary or targeted

Several perspectives were provided on how domain-specific an intervention needed to be to have relevance (from narrow to broad; e.g., for ballet, for dance, for the performing arts, for performers generally). A case could be made for either option, as described by Emily: “*I think you either go big and broad and recognize that all artists and creative people have these needs and desires in common, or you go absolutely focused and specific.*”

Ava felt that an intervention could be suited to athletes from different sports and performers from different disciplines together, as long as it is targeting the core themes of being a performer: “*It’s about struggling and striving and being able to allow yourself to be as free to engage in that forward space as you can without getting in your own way.*” Alexander also described how these courses could be suitable to athletes and performing artists in combination: “*I think just high-performance achievers is who you are looking for and I think it’s okay to combine that with sports… folks who are focused on, ‘I spend hours and hours and hours preparing for one hour… It’s prepare, prepare, prepare, and then everyone’s looking at us for 90 min’… But that’s the same for a tennis player or a swimmer or whatever. So, I think a high-performance course is great*.”

Several participants raised that it may be most beneficial with younger performers, especially in schools or training environments. Emily suggested “*I think it’s an excellent resource for everybody. But I also think particularly younger performers who have not necessarily developed the sense of self-care and resilience that they might need.*” Olivia felt it was critical for programs like this to be implemented into training: “*I would like to see some of these ideas integrated into the syllabus and the curriculum of these places where we are kind of spouting out performers…I think we have gone too long thinking that we can just focus on performance and neglect the whole wellbeing piece.*”

##### 5.2.4.2 Intervention content must vary in format and have applied relevance

Participants gave their views on the meditations and connected that to adapting future interventions. Considerable feedback was given for the structure and content of the meditations. Participants requested the option for both shorter and longer meditations. They valued variation, in terms of length, content, and voice (including perceived gender). Several mentioned the benefit of background music and placed value on high quality sound and production. One of the most frequently raised suggestions was for meditations that could be used at specific times, such as pre- or post-performance, as stated by Ava: “*as a preparation kind of practice, or if I’m thinking about a reflective practice around things that maybe have not gone well.*” This was perhaps put most clearly by Emily, who asked: “*Okay, how do we apply this?” Does this happen just before you go on stage? Is there a 30-second meditation that we can adapt for performers just before they go on, almost like an actor’s prayer?*”

Participants described the need for both theory and practice to help participants understand SC better. This involved “*helping the participant, hand-holding the participant through a whole exercise of, ‘Hey, this is what it is… This is the thought process, not this… go this way’*” (Alexander). This could be provided through different forms, as described by Ava: “*if I think of an online delivery platform, a mixture of information, videos and guided meditations.*”

Several participants identified that accessing the intervention on their phone was critical. As described already, many felt that implementing the intervention within a performing arts school, company, or organization would be most effective. The pros and cons of online vs. in-person was discussed by many participants, with the trade-off well described by Olivia: “*I do lean towards in-person typically, is my preference. However, I think I’m naive to think that just cannot scale anymore… I think whether we would prefer it or not, there is going to have to be an online, on-demand component for these things to work.*”

##### 5.2.4.3 The desire for community

Irrespective of the intervention format, participants appeared to value having a sense of connection, community, and mentorship. It was voiced that there may be ways to obtain this even through an online or app-based intervention. Ava spoke about the value of normalizing difficult experiences and the need for SC: “*One of the things that’s really beneficial about that is seeing people look around and go, ‘Oh, I’m not alone. I’m not alone with this experience. Other people are giving themselves a hard time on the inside too.’ Because in our professional space, there’s a lot of internalizing with those, ‘Well, I’m all fine and we are fine. I’m really confident on the outside,’ turning a lot of that criticism inwards.*”

“*I’d think it would be good if there was avenues where people could just talk about, and people just sharing and hearing from other people, ‘That’s exactly what I’m going through.’ And I think it’s something reassuring that you are not alone and that what you are feeling is quite natural and normal, and other people feel that way too… I’ve been always wondering whether there is some sort of support group or something like that for artists specifically. And I think nowadays something like an online, informal gatherings, that would be quite enticing if people can just sit down and chat and talk to each other about self-compassion and how they are dealing with it and mental health*” (Ethan).

Relatedly, Alexander felt that having mentors or guides to supplement an intervention would be helpful: “*had [the research team] been active, mentor-ish for us, I probably would’ve called you up and said, ‘Hey. So, how does someone have high self-efficacy and compassion?’… and you would’ve had a really good answer for it and I would’ve saved two weeks of anguish trying to figure it out*.”

## 6 Discussion

There is a strong theoretical rationale for the role of SC in supporting performing artists’ mental health and performance. Yet, to date research has only just begun to explore these relationships (Farley and Kelley, 2023; Kelley and Farley, 2019). While the two studies presented here feature important limitations that warrant significant caution regarding any applied considerations, they do provide early and preliminary evidence as to how SC can be utilized to promote mental health among performing artists. The findings across both studies point to the need for future CBIs to be developed and robustly tested within this population.

Study 1 demonstrated that among a diverse sample of performing artists, SC was associated with better mental health. Specifically, across a range of analyses controlling for stress, body appreciation, and alcohol use, the intention to be self-compassionate was consistently associated with lower depressive and anxious symptoms, and higher wellbeing. Notably however, these effects for SC were not as strong as for stress and alcohol use, which were included as important covariates given that they have previously been shown to be highly relevant in this population (Noh et al., 2009; Szabó et al., 2020). Conversely, self-compassionate intention tended to be more strongly associated with mental health outcomes than body appreciation. In this study, SC as assessed through the CMAS was more strongly associated with mental health than through the SCS. The findings are further aligned with previous research (Muris and Petrocchi, 2017) in that it was the negative, rather than positive, components of the SCS that were most associated with mental health. This has important implications for measurement of SC, and points to the need for further refinement in the measurement of SC among performers. In sum, the findings from study 1 support the rationale for further nurturing SC among performing artists as an approach for supporting mental health (Walton et al., 2022).

Subsequently, and in pursuit of working toward this intention, Study 2 provided the first preliminary examination of the role of CBI among performing artists. Findings suggested that a brief intervention comprising of a workshop and compassion-based meditations led to self-reported improvements in SC among a small self-selecting sample of performing artists. While there was no control group in this study, pre-post raw scores showed medium-sized improvements in total scores on the CMAS and SCS, with a particularly large improvement in the negative or uncompassionate subscale score (self-judgment, isolation, and over-identification) of the SCS. This is critical given that meta-analyses have shown these negative subscales are more strongly linked to poor mental health outcomes (Muris and Petrocchi, 2017). In addition to improved SC, there were smaller improvements in wellbeing, psychological distress, body appreciation, and fears of self-compassion. Mean scores on measures of acceptability, appropriateness, and feasibility were also very high. Most participants agreed with statements reflecting improved understanding, enjoyment, and interest, and every participant reported that SC was important to performing artists and that they would be interested in taking part in a larger CBI.

Qualitative themes generally reinforced these findings, suggesting performers perceived the intervention to be effective. Providing more contextual relevance, participants described how and why these interventions may be helpful. Participants tended to report that SC was a valuable resource for responding to and managing the many stressors they face in their industry. These ranged from internal traits and tendencies (e.g., self-criticism, perfectionism) to more external and environmental factors (e.g., external criticism, financial stress, bullying). Importantly however, there were several fears, blocks, and resistances to SC (Gilbert and Mascaro, 2017; Kirby et al., 2019) especially at the outset of the study. This included most prominently the concern that SC may reduce performance or standards. This is a common fear among high performers such as athletes (e.g., Zhang and McEwan, 2023; Walton et al., 2020; Ferguson et al., 2014) and alleviating these concerns should be a key focus of future interventions. In this sample, initial fears of SC appeared to be reduced by gaining a better understanding of what SC was through knowledge, self-discovery, and practice. Potentially for participants in Study 2, this increased understanding of SC and reduction in fears allowed for the other self-perceived benefits such as increased compassionate responding to stressors and improved wellbeing to occur. In future studies, it is critical to help high performers understand from the outset that contrary to common intuitions about lowering standards, self-compassion may actually be associated with performance-related benefits such as increased self-improvement motivation (e.g., Breines and Chen, 2012).

As this was a proof-of-concept study, a core focus was on obtaining data to inform how future CBIs could be most effective in this population. Here we present some of these with the aim of supporting other researchers in building interventions. In terms of target audience, participants believed that these interventions could be narrow (e.g., aimed at ballet dancers) or broader (e.g., aimed at performers more generally), as long as the intervention targeted the underlying risks and themes relevant to SC, primarily around self-criticism and performance pressures (Walton et al., 2022). Participants’ preferences tended to vary regarding the format of any future CBI, with benefits and limitations of online or in-person intervention difficult to balance. Further preferences included blending psychoeducation - especially to target fears, blocks, and resistance - with more formal intervention and practice exercises. Participants valued variation and thus there is a need for different forms and types of meditation or exercises which could be used at different times. For example, participants valued having both short and long forms, as well as content specifically directed toward difficulties immediately pre- and post-performance. Finally, there was a high value placed on community within these interventions, to validate, normalize, and learn from others. Many participants described that implementing these interventions within ongoing training such as within performing arts schools may likely deliver the most lasting impact. This is a critical next step: first in terms of comprehensive research studies, but then in application and practice. Eventually, we anticipate that the teaching of SC as a valuable resource for performing artists may be common practice within performing arts education and workplaces.

### 6.1 Limitations and future directions

There were important limitations to both studies which must be considered when interpreting the findings of this work. The primary limitation to both studies was the significant alterations made to the final sample due to fraudulent registrations. Fraudulent ‘imposter’ participants are becoming an increasing concern in online research (Pozzar et al., 2020; Ridge et al., 2023). In the current study, it is hypothesized that imposter participants were high due to the financial incentives advertised through social media in Study 1 and reimbursement advertised in Study 2. In hindsight we did not have sufficient safeguards in place (e.g., attention checks), and therefore had to rely on a rigorous post-hoc removal of suspicious participants. We acknowledge that this is a significant issue - especially in Study 2 - and could limit broader applicability of our findings. Nevertheless, the final sample in Study 1 is still larger than any other published study on SC in the performing arts to date, and the depth within Study 2’s qualitative data helps to build on many of the drawbacks of the smaller sample. To our knowledge, Study 2 is the first report in the published literature on a CBI among performing artists, and thus provides significant value to the field, albeit with precaution.

The second key limitation was the relatively non-specific and heterogeneous nature of our sample. Performing artists are an extremely diverse group, and even a relatively focused sample (dancers) will have highly heterogeneous experiences, including across sub-disciplines (e.g., contemporary, ballet). We intentionally recruited broadly rather than specifying a specific sub-population in line with our prior rationale that the performance aspect of performing artists (and athletes) is the core unifying experience making SC a relevant approach (Walton et al., 2022). While we have chosen to group the performing arts broadly to maximize potential future scalability of interventions, future studies may instead choose to prioritize more specific populations and interventions. Further, this sample were self-selecting and were clearly interested in CBIs and so potentially less resistant to these sorts of interventions. The absence of a control group further complicates the interpretation of the positive findings in Study 2, and we cannot rule out that participants may have been influenced by placebo or novelty-induced effects. Future studies with larger and more generalizable samples (such as within a performing school) are needed to further understand acceptability and efficacy while reducing where possible any potential selection bias. Given this self-selecting cohort we have no reason to suspect inaccurate diary recording of meditations. Nevertheless, the lack of an objective (i.e., digital recording) recording of meditation use is another limitation. Future studies may benefit from finding more reliable forms of assessing participant adherence in terms of both consistency and engagement. Finally, the analysis plan was not pre-registered, and this hinders transparency around the analysis. Future studies would benefit from this more robust approach.

Several important future directions are now relevant. First, and regarding Study 1, longitudinal studies exploring the potential mediating role of SC between performance-related stressors and mental health is now necessary to obtain a more complete understanding as to how SC supports performers’ health and performance. Our findings replicate other populations demonstrating that SC is highly related to various mental health outcomes. However, only longitudinal work can demonstrate how SC may mediate responses to performance-based stressors in ways that positively influence mental health. Second, Study 2 provides insight as to how CBIs can be designed to best suit performing artists. Development of CBIs for performers should take these suggestions into consideration, especially, it would seem, the need for high levels of psychoeducation, community, varied content, and clear application. Dependent on population and feasibility, research evaluation of expanded CBIs that include these factors are well justified. Given the prominence of navigating fears, blocks and resistance to SC among participants in Study 2, future CBIs must be sure to acknowledge and work with these from the beginning of the intervention (Steindl et al., 2022).

### 6.2 Conclusion

Performing artists face many difficult internal, interpersonal, and environmental stressors which can affect mental health. Resting upon a large and ever-growing body of literature in compassion science (Kirby, 2025) as well as emerging research among athletes (Cormier et al., 2023), there is a clear rational for the application of SC to help performers manage these difficulties. While acknowledging the small sample in study 2, it appears that SC may be well-placed to combat some of these issues. The preliminary findings reported here suggest that CBIs could be well received by performing artists, with our brief intervention contributing to high perceptions of acceptability, appropriateness, feasibility and potential effectiveness. There is much more to be understood about the nature of SC in the performing arts and further studies are needed to rigorously develop and examine CBIs among this population to combat the high rates of mental ill-health experienced by this group.

## Data Availability

Supporting data are not available as participants of this study were not asked to consent for their data to be shared publicly. The raw data supporting the conclusions of this article will be made available following request, without undue reservation. The analysis code and key study materials however, are available from https://osf.io/3kdsg/.
