# Supplementary material for: A preliminary investigation into self-compassion and compassion-based intervention for mental health in the performing arts
Source: Front Psychol. 2025 Feb 6;16:1512114. doi: 10.3389/fpsyg.2025.1512114 (PMC11841440; doi:10.3389/fpsyg.2025.1512114)
Supplement: Supplementary file 1 [file Table_1.docx]

Supplementary Table 1. Participant characteristics by group

|  | **Music (n=111)** | **Dance (n=68)** | **Acting (n=32)** | **Significance** |
| --- | --- | --- | --- | --- |
| **Age (Mean, SD)** | 35.7 (13.5) | 27.6 (12.1) | 33.2 (12.7) | <.001^1^ |
| ***Gender*** |  |  |  | 0.012^2^ |
| Women | 66 (59.5%) | 50 (73.5%) | 15 (46.9%) |  |
| Men | 37 (33.3%) | 10 (14.7%) | 15 (46.9%) |  |
| Non-binary or another gender | 8 (7.2%) | 8 (11.8%) | 2 (6.2%) |  |
| ***Sexual Orientation*** |  |  |  | 0.437^2^ |
| Heterosexual | 66 (59.5%) | 43 (63.2%) | 23 (71.9%) |  |
| Other | 45 (40.5%) | 25 (36.8%) | 9 (28.1%) |  |
| ***Aboriginal or Torres Strait Islander*** | 106 (95.5%) | 66 (97.1%) | 31 (96.9%) | 0.849^2^ |
| ***Current Role*** |  |  |  | 0.262^2^ |
| Paid performer | 83 (74.8%) | 47 (69.1%) | 27 (84.4%) |  |
| Student performer | 28 (25.2%) | 21 (30.9%) | 5 (15.6%) |  |
| ***Current or previous mental health diagnosis*** | 64 (57.7%) | 34 (50.0%) | 19 (59.4%) | .738^2^ |

1. Linear Model ANOVA
2. Pearson’s Chi-squared test
