# Supplementary material for: A preliminary investigation into self-compassion and compassion-based intervention for mental health in the performing arts
Source: Front Psychol. 2025 Feb 6;16:1512114. doi: 10.3389/fpsyg.2025.1512114 (PMC11841440; doi:10.3389/fpsyg.2025.1512114)
Supplement: Supplementary file 2 [file Table_2.docx]

Supplementary Table 2. Mental health and self-compassion by performer type

|  | **Music (n=111)** | **Dance (n=68)** | **Acting (n=32)** | **Significance** |
| --- | --- | --- | --- | --- |
| **Perceived stress (PSS)** |  |  |  | .002^1^ |
| Mean (SD) | 20.8 (6.9) | 24.3 (5.5) | 20.4 (4.5) |  |
| Median (Q1, Q3) | 20.5 (16.2, 26.0) | 24.0 (21.0, 28.0) | 20.5 (17.0, 24.0) |  |
| Missing | 9 | 13 | 4 |  |
| **Perceived Stress (PSS) categories** |  |  |  | .012^2^ |
| Low stress | 14 (13.7%) | 1 (1.8%) | 1 (3.6%) |  |
| Moderate Stress | 68 (66.7%) | 36 (65.5%) | 24 (85.7%) |  |
| High perceived stress | 20 (19.6%) | 18 (32.7%) | 3 (10.7%) |  |
| **Body Appreciation (BAS2)** |  |  |  | .038^1^ |
| Mean (SD) | 3.2 (0.9) | 3.0 (0.8) | 3.5 (0.9) |  |
| Median (Q1, Q3) | 3.2 (2.6, 3.9) | 2.9 (2.5, 3.5) | 3.3 (2.8, 4.2) |  |
| Missing | 6 | 13 | 2 |  |
| **Risky alcohol use (AUDIT-C)** |  |  |  | .780^1^ |
| Mean (SD) | 3.9 (2.9) | 3.7 (2.6) | 3.5 (2.5) |  |
| Median (Q1, Q3) | 4.0 (1.8, 6.0) | 3.0 (2.0, 5.0) | 3.0 (1.2, 5.8) |  |
| Missing | 11 | 8 | 2 |  |
| **Anxiety (GAD7)** |  |  |  | .002^1^ |
| Mean (SD) | 9.1 (5.9) | 12.4 (5.9) | 8.9 (5.9) |  |
| Median (Q1, Q3) | 8.0 (5.0, 13.0) | 12.0 (6.5, 18.0) | 7.0 (4.0, 13.8) |  |
| Missing | 8 | 9 | 2 |  |
| **Likely anxiety (GAD7 categories)** | 47 (45.6%) | 39 (66.1%) | 12 (40.0%) | .018^2^ |
| **Depression (PHQ9)** |  |  |  | <.001^1^ |
| Mean (SD) | 10.8 (7.0) | 14.4 (7.4) | 8.8 (6.5) |  |
| Median (Q1, Q3) | 9.0 (6.0, 15.2) | 14.0 (9.0, 20.0) | 7.0 (4.0, 12.5) |  |
| Missing | 7 | 11 | 1 |  |
| **Likely depression (PHQ9 categories)** | 47 (45.2%) | 42 (73.7%) | 11 (35.5%) | <.001^2^ |
| **Wellbeing (WEMWBS)** |  |  |  | .004^1^ |
| Mean (SD) | 43.9 (10.3) | 39.7 (9.0) | 46.9 (11.8) |  |
| Median (Q1, Q3) | 42.5 (37.0, 52.0) | 38.0 (33.0, 47.0) | 46.5 (37.8, 56.0) |  |
| Missing | 11 | 13 | 0 |  |
| **Self-Compassion (SCS)** |  |  |  | .002^1^ |
| Mean (SD) | 2.8 (0.7) | 2.5 (0.6) | 3.0 (0.6) |  |
| Median (Q1, Q3) | 2.8 (2.3, 3.2) | 2.6 (2.1, 3.0) | 3.0 (2.6, 3.5) |  |
| Missing | 12 | 12 | 3 |  |
| **SCS – Compassionate responding** |  |  |  | .060^1^ |
| Mean (SD) | 3.1 (0.8) | 2.9 (0.7) | 3.2 (0.6) |  |
| Median (Q1, Q3) | 3.0 (2.6, 3.6) | 2.9 (2.3, 3.4) | 3.2 (2.8, 3.6) |  |
| **SCS – Uncompassionate responding** |  |  |  | .003^1^ |
| Mean (SD) | 3.5 (0.8) | 3.8 (0.6) | 3.1 (1.0) |  |
| Median (Q1, Q3) | 3.6 (2.9, 4.1) | 3.8 (3.2, 4.2) | 3.5 (2.4, 3.9) |  |
| **Fears of (self-) compassion (FSCS)** |  |  |  | .005^1^ |
| Mean (SD) | 21.1 (14.2) | 28.1 (15.1) | 18.8 (14.8) |  |
| Median (Q1, Q3) | 20.0 (8.5, 31.0) | 29.5 (17.5, 40.0) | 14.0 (7.0, 32.0) |  |
| Missing | 12 | 12 | 3 |  |
| **Self-compassion (CMAS)** |  |  |  | .007^1^ |
| Mean (SD) | 86.0 (18.1) | 79.9 (18.4) | 92.7 (17.3) |  |
| Median (Q1, Q3) | 88.0 (74.2, 99.0) | 78.5 (69.0, 91.5) | 95.0 (84.0, 100.2) |  |
| Missing | 13 | 8 | 4 |  |
| **Self-compassion distress tolerance (CMAS)** |  |  |  | .003^1^ |
| Mean (SD) | 31.7 (9.2) | 29.2 (8.7) | 36.3 (7.8) |  |
| Median (Q1, Q3) | 31.0 (26.0, 39.0) | 29.0 (24.0, 35.5) | 36.0 (31.0, 42.0) |  |
| **Self-compassion intention (CMAS)** |  |  |  | .065^1^ |
| Mean (SD) | 29.0 (5.5) | 27.0 (5.8) | 29.4 (5.7) |  |
| Median (Q1, Q3) | 30.0 (26.0, 34.0) | 28.0 (22.8, 31.0) | 30.5 (25.8, 35.0) |  |
| **Self-compassion action (CMAS)** |  |  |  | .179^1^ |
| Mean (SD) | 25.3 (8.0) | 23.7 (8.3) | 27.0 (7.7) |  |
| Median (Q1, Q3) | 25.5 (22.0, 30.0) | 25.0 (19.0, 29.0) | 28.0 (22.2, 33.2) |  |

1. Linear Model ANOVA
2. Pearson’s Chi-squared test
