# Supplementary material for: A preliminary investigation into self-compassion and compassion-based intervention for mental health in the performing arts
Source: Front Psychol. 2025 Feb 6;16:1512114. doi: 10.3389/fpsyg.2025.1512114 (PMC11841440; doi:10.3389/fpsyg.2025.1512114)
Supplement: Supplementary file 3 [file Table_3.docx]

Supplementary Table 3. Imputed partially- and fully- adjusted linear regression for depression, anxiety, and wellbeing

|  | **Predictor** | **Partially adjusted** | | **Fully adjusted** | |
| --- | --- | --- | --- | --- | --- |
|  |  | **Coef (95% CI)** | **p-value** | **Coef (95% CI)** | **p-value** |
| **Depression (PHQ9)** | Perceived Stress (PSS) | 0.58 (0.47, 0.70) | <0.001 | 0.52 (0.39, 0.65) | <0.001 |
|  | Body Appreciation (BAS2)* | 0.43 (0.31, 0.55) | <0.001 | 0.14 (0.01, 0.26) | 0.031 |
|  | Risky Alcohol Use (AUDIT) | 0.36 (0.24, 0.48) | <0.001 | 0.26 (0.16, 0.36) | <0.001 |
|  | SC-Intention (CMAS)* | 0.39 (0.26, 0.52) | <0.001 | 0.18 (0.05, 0.31) | 0.009 |
|  | SC-Distress Tolerance (CMAS)* | 0.35 (0.21, 0.48) | <0.001 | -0.14 (-0.28, 0.01) | 0.070 |
| **Anxiety (GAD7)** | Perceived Stress (PSS) | 0.64 (0.53, 0.75) | <0.001 | 0.55 (0.41, 0.68) | <0.001 |
|  | Body Appreciation (BAS2)* | 0.45 (0.33, 0.57) | <0.001 | 0.12 (-0.01, 0.24) | 0.075 |
|  | Risky Alcohol Use (AUDIT) | 0.29 (0.16, 0.41) | <0.001 | 0.19 (0.09, 0.30) | <0.001 |
|  | SC-Intention (CMAS)* | 0.38 (0.26, 0.51) | <0.001 | 0.15 (0.03, 0.28) | 0.019 |
|  | SC-Distress Tolerance (CMAS)* | 0.42 (0.29, 0.55) | <0.001 | -0.04 (-0.19, 0.11) | 0.583 |
| **Wellbeing (WELWBS)*** | Perceived Stress (PSS) | 0.67 (0.55, 0.78) | <0.001 | 0.43 (0.30, 0.57) | <0.001 |
|  | Body Appreciation (BAS2)* | 0.60 (0.49, 0.71) | <0.001 | 0.30 (0.18, 0.43) | <0.001 |
|  | Risky Alcohol Use (AUDIT) | 0.21 (0.07, 0.34) | 0.003 | 0.09 (-0.01, 0.19) | 0.087 |
|  | SC-Intention (CMAS)* | 0.42 (0.29, 0.55) | <0.001 | 0.14 (0.02, 0.27) | 0.026 |
|  | SC-Distress Tolerance (CMAS)* | 0.53 (0.40, 0.65) | <0.001 | 0.03 (-0.12, 0.18) | 0.677 |
